# Reduced body weight gain in ubiquilin‐1 transgenic mice is associated with increased expression of energy‐sensing proteins

**DOI:** 10.14814/phy2.13260

**Published:** 2017-04-18

**Authors:** Fangfang Qiao, Kirsty R. Longley, Shelley Feng, Sabrina Schnack, Hongbo Gao, Yifan Li, Evelyn H. Schlenker, Hongmin Wang

**Affiliations:** ^1^ Division of Basic Biomedical Sciences and Center for Brain and Behavior ResearchSanford School of MedicineUniversity of South DakotaVermillionSouth Dakota

**Keywords:** Body weight, energy‐sensing proteins, mice, ubiquilin‐1

## Abstract

Ubiquilin‐1 (Ubqln1), a ubiquitin‐like protein, is implicated in a variety of pathophysiological processes, but its role in mediating body weight gain or metabolism has not been determined. Here, we demonstrate that global overexpression of Ubqln1 in a transgenic (Tg) mouse reduces the animal's body weight gain. The decreased body weight gain in Tg mice is associated with lower visceral fat content and higher metabolic rate. The Ubqln1 Tg mice exhibited reduced leptin and insulin levels as well as increased insulin sensitivity manifested by homeostatic model assessment of insulin resistance. Additionally, the reduced body weight in Tg mice was associated with the upregulation of two energy‐sensing proteins, sirtuin1 (SIRT1) in the hypothalamus and AMP‐activated protein kinase (AMPK) in the skeletal muscle. Consistent with the in vivo results, overexpression of Ubqln1 significantly increased SIRT1 and AMPK levels in the mouse embryonic fibroblast cell culture. Thus, our results not only establish the link between Ubqln1 and body weight regulation but also indicate that the metabolic function of Ubqln1 on body weight may be through regulating energy‐sensing proteins.

## Introduction

The maintenance of an adequate body weight is crucial for the health and survival of humans and other mammals. Body weight stabilizes if food intake is equal to energy expenditure (Jequier and Tappy 1999). When energy intake surpasses energy expenditure, the body can store the excess energy as body fat in the form of triglyceride in adipose tissue (Galgani and Ravussin 2008). Chronic excess body fat accumulation leads to obesity, a metabolic disorder that has negative effects on many organs (Smith et al. 2014), resulting in reduced life expectancy and increased risk of developing serious health problems such as heart disease, high blood pressure, type 2 diabetes (Chavali et al. 2013), cancer, psychiatric problems (Simon et al. 2006), and impaired cognitive functions (Davidson et al. 2014; Nguyen et al. 2014). An opposite clinically relevant problem resulting in loss of body weight (or cachexia) is associated with many disorders such heart failure, chronic obstructive pulmonary disease and cancer (Ebner et al. 2013; Petruzzelli and Wagner 2016).

Energy balance and body weight are regulated by complex interactions of signals from peripheral organs and the central nervous system (Chaptini et al. 2010). The hypothalamus is thought to be the master regulator of endocrine system and can sense and integrate both humoral and neural signals from the periphery to control body weight and energy balance (Lin et al. 2014). Dysregulation of hypothalamus function is involved in metabolic disorders such as obesity and its major complication type 2 diabetes (Helfer and Tups 2016). In addition, peripheral tissue‐derived hormones can be influenced by genetic factors, gene‐gene and gene‐environmental interactions (Mutch and Clement 2006). Leptin and insulin are two adiposity signals that act on the brain to regulate food intake and energy balance (Benoit et al. 2004). Leptin, an adipocyte‐derived hormone, circulates in the blood and binds to leptin receptors in the hypothalamus and other brain areas to suppress appetite and food intake and enhance energy expenditure (Morris and Rui 2009). Insulin, a hormone produced by the *β* cells in the pancreas, helps maintain blood glucose balance by regulating the metabolism of sugar and fat. The leptin and insulin levels in the blood are positively correlated with adipose mass (Benoit et al. 2004).

Sirtuin1 (SIRT1) and AMP‐activated protein kinase (AMPK) are two metabolic energy sensors (Ruderman et al. 2010). SIRT1 is an NAD^+^ (nicotinamide adenine dinucleotide)‐dependent histone/protein deacetylase and is widely expressed in mammalian cells (Sinclair and Guarente 2006). It is involved in mediating fat and glucose levels related to alteration in energy homeostasis (Houtkooper et al. 2012). AMPK, a metabolic sensor, is activated by an elevated cellular AMP/ATP ratio. Activation of SIRT1 and AMPK is associated with the stimulation of catabolic pathways and inhibition of anabolic pathways (Ruderman et al. 2010; Hardie et al. 2012). Since SIRT1 and AMPK are closely related to energy expenditure (Cantó and Auwerx 2009), they may be potential biomarkers to evaluate cellular energy balance.

Ubiquilin 1 (Ubqln1, previously also referred to as PLIC‐1) is a ubiquitin‐like protein that belongs to the Ubqln family containing at least four members in humans and rodents (Marin 2014). Ubqln1 is implicated in protein degradation pathways including both proteasome and autophagy pathways (Kleijnen et al. 2000; Ko et al. 2004; Rothenberg et al. 2010). Because protein quality control plays an important role in maintaining cellular proteostasis, dysregulation of Ubqln1 is involved in multiple pathophysiological conditions. Previous reports have mainly linked Ubqln1 to nutrient starvation and autophagy (N'Diaye and Brown 2003), neurodegenerative diseases including Alzheimer's disease (Mah et al. 2000; Bertram et al. 2005; Hiltunen et al. 2006; Stieren et al. 2011; Viswanathan et al. 2011, 2013; el Ayadi et al. 2012; Zhang and Jia 2014; Yue et al. 2015) and Huntington's disease (Wang et al. 2006; Safren et al. 2014). Currently, only a few of studies examined the function of Ubqln1 in animal models and human with Alzheimer's disease (Li et al. 2007; Stieren et al. 2011; Mizukami et al. 2014), Huntington's diseases (Wang et al. 2006; Safren et al. 2014), and oxidative stress and ischemic stroke‐caused injury (Liu et al. 2014a). Due to its crucial role in the protein quality control, Ubqln1 may also be involved in other signaling pathways affecting body weight regulation. The relationship between Ubqln1 and body weight has not been investigated in animal models. Our study for the first time suggests that Ubqln1 might be an important factor affecting the complex body weight‐regulating system.

## Materials and Methods

### Animals

The Tg mice overexpressing Ubqln1were originally generated in a C57BL6/SJL hybrid background (Liu et al. 2014b). Compared to Wt mice, the Ubqln1 Tg mice exhibited stronger overexpression of Ubqln1 in the brain, heart, liver, and muscle compared with other tissues (Liu et al. 2014b). After being crossed with the C3H/B6 hybrid background mice, at 3 months the Tg mice showed about 10% lighter body weight than their Wt littermates. To confirm this phenotype, the Tg mice were further crossed with C3H/B6 mice and the offspring mice were characterized in this study. The C3H/B6 mice were obtained from the Jackson laboratory (Bar Harbor, Maine).

All animal maintenance and experimental procedures performed were in accordance with the National Institute of Health Guide for the Care and Use of Laboratory Animals and were approved by the Institutional Animal Care and Use Committee of the University of South Dakota. Mice were maintained in a temperature and humidity controlled environment with a 12‐h/12‐h light/dark cycle. The animals had ad libitum access to food and water. Both male and female mice were used in this study.

### Measurement of body weight

Male and female mice were separately housed with a maximum of four animals a cage. Animals were fed a regular chow (energy density 3.1 kcal/g) with 24% calories from protein, 16% calories from fat and 60% calories from carbohydrate (2920X, ENVIGO, Indianapolis, IN). Body weights of mice were determined monthly starting at 1 month of age.

### Measurement of organ weights and visceral fat

All mice were sacrificed at 18 months of age. The brain, liver, heart, lung, spleen, kidney, and visceral fat from each mouse were collected and immediately weighed. Data are presented as absolute organ weights and visceral fat weight and the ratio of visceral weight to body weight.

### Physiological analysis

*Body temperature* was measured in 17 months old mice during the day (9–12 pm) and at night (8–11 pm) with a model BAT‐12 thermometer connected to a thermoprobe. Prior to insertion into the rectum the probe covered with mineral oil. The probe was inserted about 1 cm into the rectum of the mouse to measure the body temperature. Probes were cleaned following each measurement.

*Metabolic rate* was determined in 17 months old mice using indirect calorimetry during the day (9–12 pm) and at night (8–11 pm) as detailed below. Before studies commenced, each mouse was weighed. All the tests were performed at room temperature (22–25°C). The mice were kept in the test room for 30 min prior to being placed into the 8.5 cm long 6.5 cm diameter Plexiglas cylindrical chamber. Without a mouse in the chamber and air flowing through the chamber oxygen (O_2_) entering (FIO_2_) and carbon dioxide (CO_2_) entering (FICO_2_) were measured. Flowrates through the chamber were measured using a rotameter and averaged 1 L/min. Subsequently the mouse was placed into the chamber with air flowing through it, acclimated for 5 min, and then the fraction of expired O_2_ (FEO_2_) and CO_2_ (FECO_2_) were determined. The fractional contents of O_2_ and CO_2_ were measured with an O_2_ analyzer (VacuMed Fast Response Analyzer) and a CO_2_ analyzer (VacuMed Golden Edition Analyzer), respectively. Following the measurements, the mouse was removed from the chamber. Prior to testing the next animal, the chamber was cleaned with 70% alcohol. O_2_ consumption and CO_2_ production for each mouse were calculated using the following equations: O_2_ consumption (*V*O_2_) = (25*Flowrate‐152)*(FIO_2_‐FEO_2_) and CO_2_ production (*V*CO_2_) = (25*Flowrate‐152)*(FECO_2_‐FICO_2_). The respiratory quotient (RQ) = *V*CO_2_/VO_2_. Both *V*CO_2_ and *V*O_2_ were corrected by body weight.

*Plasma leptin and insulin levels* were measured in 12.5 months old mice using mouse leptin ELISA (enzyme‐linked immunosorbent assay) kits and ultrasensitive mouse insulin ELISA kits (Crystal Chem Inc., Downers Grove, IL) according to the manufacturer's instructions. The blood was collected from a tail incision into a tube containing heparin (1 unit/mL), and centrifuged for 20 min at 2000*g*. The supernatant (plasma) was used for ELISA.

### Generation of stable mouse embryonic fibroblasts from Wt and Tg mice

Primary mouse embryonic fibroblasts (MEFs) from mouse embryos were isolated from a female Ubqln1 Tg mouse bred with a Wt male mouse at embryonic day 14 according to a previously described protocol (Xu 2005). PCR was conducted for genotype identification. MEFs were maintained in a complete medium containing Dulbecco's modified Eagle medium supplemented with 10% (v/v) fetal bovine serum and 100 *μ*g/mL penicillin/streptomycin. Primary MEFs were immortalized by stable transfection of the SV40 T antigen.

### Western blot analysis

Hypothalamus isolated from the brain and the gastrocnemius muscle were collected from Wt and Tg mice for Western blot analysis at 18 months of age. In addition, stable MEFs derived from Wt and Tg mice were harvested for Western blot analysis. Immunoblotting was conducted as previously described (Lü and Wang 2012). Primary antibodies consisted of Ubqln1 (1:1000, Abcam, Cambridge, MA), SIRT1 (sirtuin1) (1:250 Cell Signaling, Danvers, MA), AMPK*α* (AMP‐dependent protein kinase‐*α*) (1:1000, Cell Signaling), GAPDH (1:1000, Cell Signaling) and Actin (1:1000, Santa Cruz). Appropriate secondary antibodies used were conjugated with fluorescent dyes (LI‐COR Inc., Lincoln, NE). The bands were detected using an Odyssey scanner (LI‐COR) and quantified using UN‐SCAN‐IT gel6.1 software (Silk Scientific Inc., Orem, Utah).

### Statistical analysis

Body weight data over time were evaluated by determining the area under the curve (AUC) for each animal. Correlations were computed using Pearson correlation coefficients (r). Values for HOMA‐IR were calculated using the following formula: fasting blood glucose (mg/dl) × fasting insulin (*μ*U/mL)/405 (Matthews et al. 1985). Statistical comparisons between two groups were evaluated using unpaired two‐tailed Student's *t*‐test. All numerical data were presented as mean ± SEM. Significance was accepted at *P* < 0.05. Data analysis was performed with Microsoft Excel 2013 and GraphPad Prism Statistical Software version 6.05.

## Results

### Ubqln1 Tg mice showed reduced body weight gain

**Figure 1:**
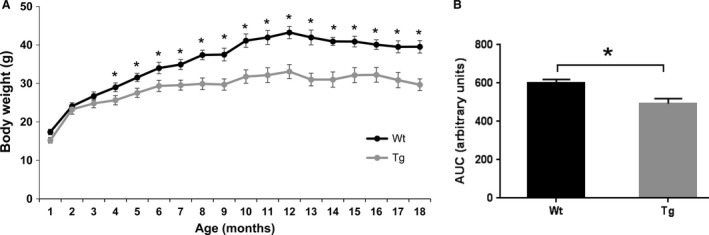
Overexpression of Ubqln1 reduces body weight gain. (A) Temporal changes in body weight of Tg mice and their Wt littermates from 1 to 18 months of age. (B) Area under the curve (AUC) of body weight was calculated for Wt and Tg mice. Wt, *n* = 10; Tg, *n* = 7. Data are shown as mean ± SEM. *P < 0.05.

The pattern of body weight with age in the two genotypes is shown in Figure 1A. The body weights in the Tg mice were significantly less than Wt mice from 4 months to 18 months. Quantification of body weight in Wt and Tg mice over time was determined by integration of area under the curve (AUC). Body weights of Tg mice were significantly less than their Wt littermates (Fig. 1B). These results suggest that overexpression of Ubqln1 suppresses the animal's body weight gain with age.

### Reduced amount of visceral fat in Ubqln1 Tg mice

**Figure 2:**
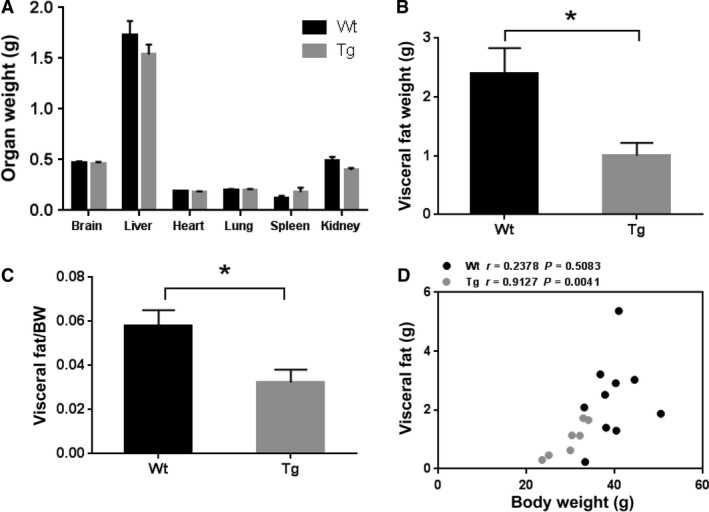
Ubqln1 Tg mice show reduced amount of visceral fat accumulation compared to their Wt littermates. (A) Measurement of absolute organ weights of 18‐month Wt and Tg mice. (B) Measurement of visceral fat weight of 18‐month Wt and Tg mice. (C) Visceral fat weight‐to‐body weight ratios of 18‐month Wt and Tg mice were calculated. (D) Correlations of body weight and visceral fat for Wt and Tg mice. BW: body weight. Wt, *n* = 8; Tg, *n* = 7. Data are shown as mean ± SEM. *P < 0.05.

To determine whether lower organ weights contributed to the diminished body weight gain in the Tg mice, we measured the absolute weights of the brain, heart, lung, liver, spleen, and kidney in the Tg mice and age‐matched Wt mice at 18 months of age. No difference in absolute organ weights between the two groups of mice was noted (Fig. 2A). However, the absolute visceral fat weights as well as the visceral fat‐body weight ratio of 18 months old Tg mice were significantly less than those of their age‐matched Wt littermate controls (Fig. 2B and C). In addition, there was a significant positive correlation between body weight and visceral fat in Tg mice (*r* = 0.9127, *P* = 0.0041) (Fig. 2D), but not in Wt mice. These data suggest that the reduced body weight gain in the Tg mice may be associated with reduced abdominal fat accumulation.

### Ubqln1 Tg mice showed higher energy expenditure

Body weight is determined by a balance between food intake and energy expenditure. We measured food intake of Wt and Tg mice at different time points. The average amount of food intake was only significantly less in the Tg mice than that in Wt controls at 2.5 and 6.5 months of age (data not shown). After 7.5 months, food intake was similar between the two groups of animal (data not shown). Reduced food intake was only observed at young ages but not older ages in Tg mice, suggesting that food intake might not be a major factor affecting differences in body weight between the two groups.

**Figure 3:**
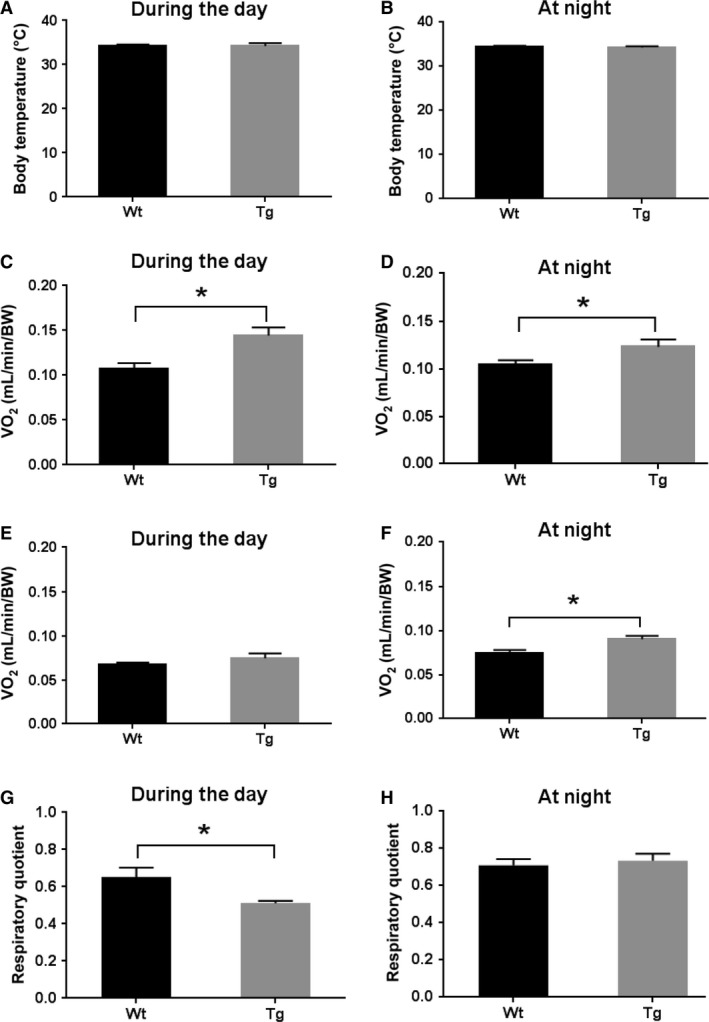
Overexpression of Ubqln1 does not change body temperature but significantly increases metabolic rates in mice. Measurement of body temperature of Wt and Tg mice at 17 months of age during the day (A) and at night (B). O_2_ consumption of 17‐month Wt and Tg mice during the day (C) and at night (D). CO_2_ production of 17‐month Wt and Tg mice during the day (E) and at night (F). Respiratory quotient (RQ) of 17‐month Wt and Tg mice during the day (G) and at night (H). BW: body weight. Wt, *n* = 10; Tg, *n* = 7. Data are shown as mean ± SEM. *P < 0.05.

Higher energy expenditure and body temperature could also account for the reduced body weight in Tg mice. At 17 months of age when body weights were substantially less in the Tg mice, body temperature did not differ between Wt and Tg mice during the day or at night (Fig. 3A and B). To examine whether lean phenotype in Tg mice is related to metabolic rate, indirect calorimetry was performed to assess metabolic rate during resting state in 17‐month‐old animals. Tg mice consumed more O_2_ (relative to their body weight) both during the day and at night than Wt animals (Fig. 3C and D). Moreover, the nighttime CO_2_ production (relative to their body weight) of the Tg mice was greater than that of their Wt counterparts (Fig. 3F). Thus, the metabolic rate of Tg mice at night was enhanced evidenced by the increased O_2_ consumption and CO_2_ production. Interestingly, the CO_2_ production of Tg mice during daytime did not differ from that determined in the corresponding Wt mice (Fig. 3E).

Although the respiratory quotient (RQ, the ratio of *V*CO_2_ to *V*O_2_), a measure of fuel source, was not different between Wt and Tg mice at night (Fig. 3H), the Tg mice showed lower RQ during the day (Fig. 3G) indicating enhanced fat oxidation (Simonson and Defronzo 1990). Collectively, these results indicated that Tg mice have increased energy expenditure compared to that of Wt mice at night that may contribute to the reduction of body weight and possibly diminished visceral fat mass in Ubqln1 Tg mice.

### Overexpression of Ubqln1 affects plasma leptin and insulin levels and HOMA‐IR in mice

**Figure 4:**
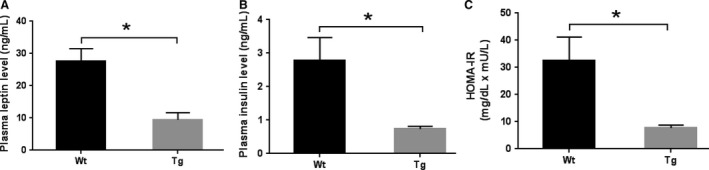
Overexpression of Ubqln1 affects plasma insulin and leptin levels and HOMA‐IR in mice. (A) ELISA analysis of plasma leptin levels in Wt and Tg mice at 12.5 month of age. (B) ELISA analysis of plasma insulin levels Wt and Tg mice at 12.5 month of age. (C) The values of HOMA‐IR were calculated for Wt and Tg mice at 12.5 months of age. Wt, *n* = 11; Tg, *n* = 9. Data are shown as mean ± SEM. *P < 0.05.

Leptin is a hormone secreted by adipose tissue and helps regulate energy balance by inhibiting appetite and stimulating energy expenditure (Kwon et al. 2016). Consistent with the reduced visceral fat mass, plasma leptin levels in Tg mice were reduced relative to Wt littermates at 12.5 months (Fig. 4A). Interestingly, the plasma insulin levels were remarkably decreased in Tg animals compared with Wt controls at 12.5 months (Fig. 4B). HOMA‐IR, the ratio of fasting glucose and insulin levels, was used to quantify insulin resistance. The data showed that the values of HOMA‐IR in Tg mice were significantly lower than that of corresponding Wt mice at 12.5 months (Fig. 4C), suggesting enhanced insulin sensitivity in Tg mice.

### Overexpression of Ubqln1 upregulate energy‐sensing proteins in the hypothalamus, skeletal muscle and MEFs

**Figure 5:**
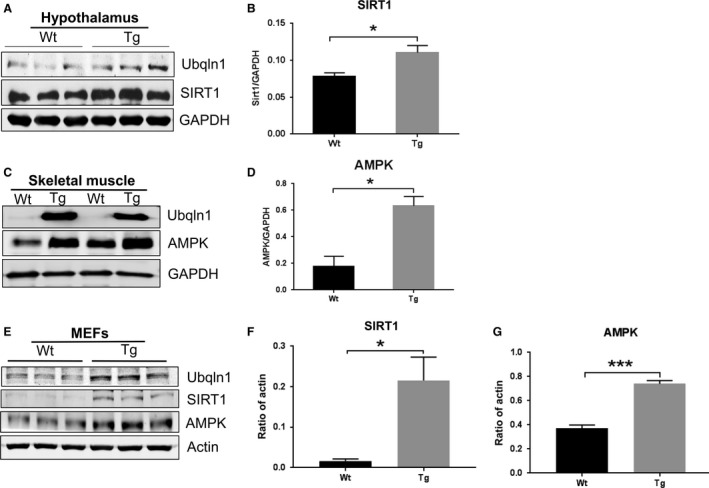
Overexpression of Ubqln1 increases energy balance relevant proteins in vivo and in vitro. (A) Western blot analysis of SIRT1 protein levels in the hypothalamus of Wt and Tg mice at 18 months. (B) Quantitative analysis of SIRT1 protein levels in the hypothalamus of Wt and Tg mice. (C) Western blot analysis of AMPK protein levels in the gastrocnemius muscle of Wt and Tg mice at 18 months. (D) Quantitative analysis of AMPK protein levels in the skeletal muscle of Wt and Tg mice. *N* = 3 or 4 for each group. (E) Western blot analysis of SIRT1 and AMPK protein levels in Wt and Tg mouse embryonic fibroblasts (MEFs). (F) Quantitative analysis of SIRT1 protein levels in MEFs from Wt and Tg mice. (G) Quantitative analysis of AMPK protein levels in MEFs from Wt and Tg mice. GAPDH or Actin was used as a loading control. Data are shown as mean ± SEM. *P < 0.05.

The hypothalamus plays an essential role in modulating energy homeostasis by integrating various signals from peripheral and central sources to influence food intake and expenditure. On the other hand, skeletal muscle is a major metabolic tissue implicated in energy consumption. To investigate how overexpression of Ubqln1 may modulate body weight and energy balance, we isolated the hypothalamus and skeletal muscle (gastrocnemius) from Wt and Tg mice at 18 months of age and performed Western blot analysis of several energy‐sensing molecules. As shown in Figure 5A and C, relative to Wt mice, Ubqln1 expression was greater in both the hypothalamus and skeletal muscle of Tg mice. Additionally, the protein level of SIRT1, an energy‐sensing protein, in the hypothalamus of Tg mice was significantly higher than that in the Wt mice at 18 months (Fig. 5A and B). Moreover, the protein level of AMPK (AMP‐activated protein kinase), a master metabolic regulator, was significantly upregulated in the skeletal muscle of Tg mice (Fig. 5C and D). These results raise the possibility that overexpression of Ubqln1 suppresses the mouse body weight gain via upregulating hypothalamic SIRT1 and skeletal muscle AMPK.

Additionally, immortalized Wt and Tg MEF (mouse embryonic fibroblast) cell lines from mouse embryos were generated. Similar to the in vivo data, SIRT1 and AMPK, were also increased in MEFs from Tg relative to Wt mice (Fig. 5E, F, and G), suggesting that the impact of overexpression of Ubqln1 on energy‐sensing proteins may have occurred in early embryo development.

## Discussion

We here demonstrate that overexpression of Ubqln1 reduces body weight gain in a specific hybrid background (C3H/B6) mice. The Ubqln1 Tg mice exhibited lower visceral fat levels, higher energy output, and reduced plasma leptin and insulin levels as compared to the Wt mice.

Additionally, we identified two upregulated energy‐sensing proteins SIRT1 and AMPK in the Tg mice and MEFs from embryonic Tg mice, which may provide clues for understanding the Tg mouse phenotypes. These results indicate that overexpression of Ubqln1 may modulate body weight gain via energy metabolism and relevant proteins.

Recent data indicated that increased body mass causes the accumulation of ubiquitinated proteins and reduced autophagy markers in the hypothalamus, whereas obesity‐resistant mutant mice show reduced ubiquitinated protein levels (Ignacio‐Souza et al. 2014). Since Ubqln1 selectively enhances unwanted protein degradation of proteasome and autophagy pathways (Wang and Monteiro 2007), it is possible that overexpression of Ubqln1 may reduce accumulation of misfolded proteins and thereby contribute to suppressing body weight gain in Tg mice (Fig. 1).

In addition to visceral fat, no differences in absolute organ weights of brain, heart, lung, liver, spleen, and kidney were noted (Fig. 2A). Not only the absolute visceral fat weight but also the visceral fat to body weight ratio were significantly decreased in Ubqln1 Tg mice (Fig. 2B and C). Thus, compare to other organs, visceral fat content is a major contributor to the reduced body weight. Whether changes in skeletal muscle mass may contribute to the reduced body weight needs to be investigated in the future.

The close association between visceral fat and body weight was also manifested in the correlation analysis result (Fig. 2D), especially in the Tg animals. Adipose tissue plays multiple metabolic and endocrine functions by secreting an array of hormones (adipokines) to maintain whole‐body metabolism (Coelho et al. 2013). Overexpression of Ubqln1 may regulate body weight by affecting the levels of adipose‐secreted hormones. To support this possibility, our results showed that the plasma levels of leptin, an adipose‐secreted hormone that exerts anorexic effects, significantly reduced in Ubqln1 Tg mice (Fig. 4A). In addition, an early study reported the new role of autophagy in mediating lipid metabolism (macrolipophagy) (Singh et al. 2009). Specifically, inhibition of autophagy leads to the increased triglyceride storage in lipid droplets, which raises the possibility that overexpression of Ubqln1 may decrease the visceral fat mass by enhancing autophagy activity and facilitating fatty acid degradation.

Reduced food intake was only observed at young ages but not older ages in Tg mice (unpublished observations) suggesting that this is not a major factor affecting differences in body weights between the two groups. There was no difference in body temperature between two groups of mice (Fig. 3A and B) indicating similar levels of heat dissipation. Based on our data, the elevated metabolic rate at night may be a major contributor of the reduced body weight in Tg mice. Moreover, the reduced RQ of Tg mice (Fig. 3G) suggests that the increased metabolism in Tg mice may result from their greater fat oxidation, which is consistent with the visceral fat results (Fig. 2). Mice are usually active during the night, but no statistical difference in O_2_ consumption in Tg mice at night versus during the day was observed in our study (repeated paired t‐test, data not shown). This may be due to our measurement was conducted at one time point and mice were in a restrained condition. The increased O_2_ consumption at night in Tg mice might be observed if the metabolism measurements were followed up for a continuous 8‐hour period at night. A study indicated that O_2_ consumption peaks in C57BL/6 mice around 7 pm (Shah et al. 2015), so the time period we selected at night (8–11 pm) may also contribute to the observation.

**Figure 6:**
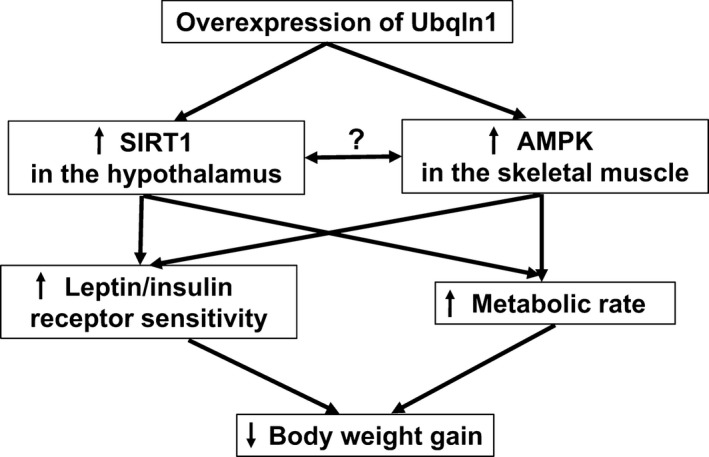
A proposed hypothetical model underlying the action of overexpression of Ubqln1 on reduced body weight gain in mice. Overexpression of Ubqln1 upregulates two energy‐sending proteins, SIRT1 and AMPK in the hypothalamus and skeletal muscle, respectively. We cannot exclude the possibility that the two energy‐sending proteins influence each other. Increased SIRT1 and AMPK may synergistically enhance leptin/insulin receptor sensitivity and elevate metabolic rate, leading to reduced body weight gain in Tg mice.

In the present study, the basal insulin level was positively correlated with body adiposity (Benoit et al. 2004) and visceral adiposity is causally associated with insulin resistance (Lebovitz and Banerji 2005). Thus, the decreased blood insulin level in Tg mice may be related to the reduced visceral fat. Moreover, our data showed that HOMA‐IR, the index of insulin resistance, of Wt mice was 4 times higher compared to that of Tg mice (Fig. 4C) suggesting that Wt mice may be compromised by insulin resistance and that the enhanced insulin sensitivity in the Tg mice may be related to overexpression of Ubqln1. In addition, since activation of leptin and insulin signaling pathways is dependent on binding of these ligands to their respective receptors, there may be an alteration of hypothalamic insulin and leptin receptors due to Ubqln1 overexpression in the Tg mice, which may increase their sensitivity (Fig. 6). Further studies evaluating the levels and function of hypothalamic insulin and leptin receptors and downstream pathways are needed to investigate these possibilities.

SIRT1 is an energy‐sensing molecule that may be responsible for promoting healthy longevity (Gao et al. 2016) through mechanisms possibly similar to caloric restriction (Chen et al. 2008). It plays an important role in body weight and metabolism regulation and suppresses body weight gain (Bordone et al. 2007; Banks et al. 2008; Pfluger et al. 2008; Cakir et al. 2009; Sasaki 2015). Loss of SIRT1 function in the hypothalamus results in leptin resistance and decreases energy expenditure contributing to diet‐induced obesity (Ramadori et al. 2010; Sasaki et al. 2014). Moreover, increased hypothalamic SIRT1 levels inhibit age‐associated weight gain by increasing leptin sensitivity in mice (Sasaki et al. 2014). It is possible that the beneficial effects conferred by the overexpression of Ubqln1 on body weight occurs in part by upregulation of SIRT1 proteins in the hypothalamus.

AMPK, as a sensor of cellular energy status, positively modulates catabolic pathways like glucose uptake, fatty acid oxidation, glycolysis, and negatively modulates anabolic pathways like lipid and protein synthesis (Hardie 2011). The increased AMPK noted in this study in skeletal muscle may also confer a protective effect on body weight gain in Tg mice. AMPK and SIRT1 are activated in responses to nutrient deficiency. Recently, multiple studies support the notion that these two energy‐sensing molecules, AMPK and SIRT1, not only share common target proteins (such as PGC1*α*, FOXO, NFKB, eNOS) and biological functions but also activate each other (Ruderman et al. 2010). It is possible that the upregulation of SIRT1 activity in hypothalamus and AMPK activity in skeletal muscle synergistically work to induce the reduced body weight gain in Tg mice (Fig. 6).

MEFs are usually used as powerful tools to study molecular mechanisms. In the Wt and Tg MEF cell lines, we also observed the upregulation of both AMPK and SIRT1 (Fig. 5E, F and G), which is consistent with the in vivo data. Upregulation of SIRT1 and AMPK in the MEFs supports the possibility that overexpression of Ubqln1 may affect the expression of energy‐sensing proteins during early development. Further investigation of the relationships between Ubqln1and SIRT1 and AMPK with age may help us better understand how Ubqln1 modulates body weight gain in the animal.

Importantly, we observed that the genetic background for Ubqln1overexpression may affect body weight. For example, when the Tg mice were repeatedly bred with C57BL/6 mice, we did not observe differences in body weight with age (Liu et al. 2014b). However, when the Tg mice were continuously crossed with the C3H/B6 hybrid background Wt mice, we obtained the Tg mice with the lower body weight observed in this study. In addition, in the follow‐up study of the same background animals, we still observed the same phenotype in Tg mice at 4, 5, and 6 months of age (unpublished observations). Previous studies have indicated that there are inherent differences between mouse strains associated with body weight gain (Montgomery et al. 2013). In addition, in response to high‐fat diet, different mouse strains showed varying susceptibility to high‐fat diet induced obesity, glucose intolerance, and insulin resistance (Montgomery et al. 2013). A genetic strain difference was also found in protein metabolism and muscle hypertrophy in rat skeletal muscle after isometric resistance training (Kobayashi et al. 2012).

In conclusion, we show that overexpression of Ubqln1 in mice induces reduction in body weight in Tg mice in a specific hybrid background (C3H/B6) relative to the age‐matched Wt mice. The Tg mice exhibited lower visceral fat content, higher metabolic rate, reduced plasma leptin and insulin levels, and upregulation of energy‐sensing proteins in different tissues compared to the age‐matched Wt mice when examined at a late stage. Increased energy‐sensing proteins in MEFs suggest Ubqln1 may be effective at early development. Additional studies investigating when the visceral fat content, metabolic, and hormone levels occur at various ages will further our understanding of the role of Ubqln1 in this genotype. How Ubqln1 overexpression affects mice fed a high‐fat diet may shed novel light on developing novel therapeutic strategies for obesity and its profound detrimental health problems.

## Conflict of Interest

The authors have no conflicting or financial interest to disclose.
